# The Temporal Order of Genetic and Pathway Alterations in Tumorigenesis

**DOI:** 10.1371/journal.pone.0027136

**Published:** 2011-11-01

**Authors:** Moritz Gerstung, Nicholas Eriksson, Jimmy Lin, Bert Vogelstein, Niko Beerenwinkel

**Affiliations:** 1 Department of Biosystems Science and Engineering, ETH Zurich, Basel, Switzerland; 2 Swiss Institute of Bioinformatics (SIB), Basel, Switzerland; 3 Department of Statistics, University of Chicago, Chicago, Illinois, United States of America; 4 Ludwig Center and Howard Hughes Medical Institute, Sidney Kimmel Comprehensive Cancer Center at Johns Hopkins, Baltimore, Maryland, United States of America; Ohio State University Medical Center, United States of America

## Abstract

Cancer evolves through the accumulation of mutations, but the order in which mutations occur is poorly understood. Inference of a temporal ordering on the level of genes is challenging because clinically and histologically identical tumors often have few mutated genes in common. This heterogeneity may at least in part be due to mutations in different genes having similar phenotypic effects by acting in the same functional pathway. We estimate the constraints on the order in which alterations accumulate during cancer progression from cross-sectional mutation data using a probabilistic graphical model termed Hidden Conjunctive Bayesian Network (H-CBN). The possible orders are analyzed on the level of genes and, after mapping genes to functional pathways, also on the pathway level. We find stronger evidence for pathway order constraints than for gene order constraints, indicating that temporal ordering results from selective pressure acting at the pathway level. The accumulation of changes in core pathways differs among cancer types, yet a common feature is that progression appears to begin with mutations in genes that regulate apoptosis pathways and to conclude with mutations in genes involved in invasion pathways. H-CBN models provide a quantitative and intuitive model of tumorigenesis showing that the genetic events can be linked to the phenotypic progression on the level of pathways.

## Introduction

Cancer progression is an evolutionary process that is driven by mutations and clonal expansions in a cell population. Mutations in cancer-associated genes can alter the behavior of a cell and result in loss of cooperation and increased proliferation. Cells with advantageous mutations eventually outgrow competing cells and tumor development proceeds by successive clonal expansions. In each clonal expansion, additional mutations are fixed in the population. Cancer progression is therefore characterized by the accumulation of these genetic changes [1], [2], [3].

Many oncogenes and tumor suppressor genes have been identified that contribute to tumorigenesis when activated or inactivated by mutation, respectively [4]. It is believed that cells need to acquire certain key properties, including those sometimes referred to as the hallmarks of cancer, to form a tumor [5], [6]. Among these functional changes are avoidance of apoptosis, angiogenesis, limitless replication potential, and invasion. Biological functions are usually maintained by one or several groups of genes that interact in functional pathways. Many signaling pathways have been identified that play a key role in carcinogenesis [7] and recently a set of twelve core pathways was defined [8], [9], [10].

Mathematical modeling of carcinogenesis has a long history, starting with multi-stage models for the interpretation of cancer incidence data [11], [12], [13]. Because cancer is an evolutionary process, population genetics has provided useful models for describing the dynamics of cancer cell populations [14], [15], [16], [17]. The order in which genetic events tend to fixate in tumors is of particular interest, because it might elucidate the critical events in carcinogenesis and could even have therapeutic applications. Tumors progress through a sequential series of genetic alterations, but the order of these alterations can vary among tumors and even among different compartments of the same tumor [8], [9], [10], [14], [17], [18], [19], [20]. This observation has prompted the development of statistical methods that generalize the assumption of a linear order in different ways [21], [22], [23], [24], [25], [26]. Most of these models have been applied to CGH data from various cancer types but not yet to detailed cancer sequencing data.

Here, we use a class of graphical models, called Hidden Conjunctive Bayesian Networks (H-CBNs) to describe the progression of cancer at the level of genes and pathways [27], [28], [29], [30], [31]. H-CBNs model the accumulation of alterations under partial order constraints allowing for small deviations in the actual data from the most likely progression model. The notion behind the partial order assumption is that there exist constraints on the sequence of genetic events characterizing the progression of cancer development for some mutations, but not necessarily for all. While in each tumor a specific linear series of mutations occurs, progression may be different among tumors. The partial order is the set of order constraints, or relations, that holds for all tumors. Here and in the following, we use the terms ‘order constraint’ and ‘relation’ synonymously. Inferring these partial order constraints from experimental data is the main aim of this study.

**Figure 1:**
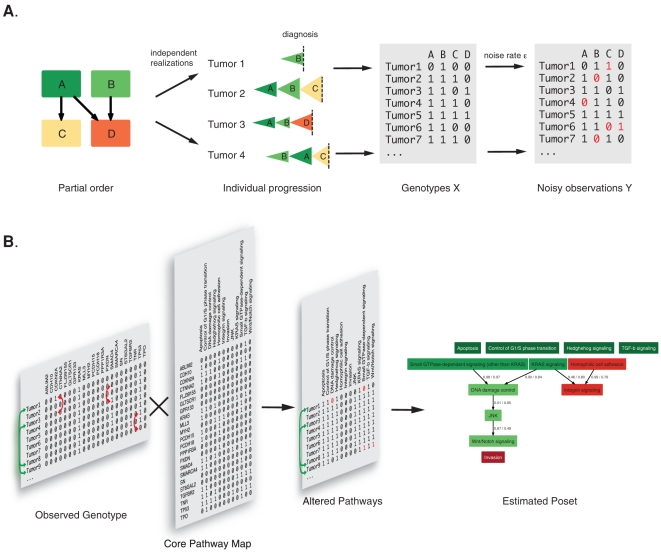
Schematic illustration of the H-CBN model and gene-to-pathway mapping. A. Partial order constraints, as denoted by arrows, restrict the possible ordering in which mutations occur. In this example, mutation C arises only after A, and mutation D requires A and B to be present. Mutations A and B can occur in any order. Because the order is only partial, the sequence of events can differ between tumors. The accumulation of each mutation is described by a stochastic exponential waiting time process that corresponds to a clonal expansion. Each tumor thus arises by a series of expansions that differs across tumors depending on the number of constraints. No constraints imply that all orderings are possible; a linear (total) ordering corresponds to a single sequence of events for all cases. Tumors are examined at diagnosis and its genotype *X* indicates all functionally altered genes that have accumulated until then (1: altered, 0: functional). The observed list of mutated genes *Y*, however, can contain errors (red) due to incomplete data or wrong interpretation of the results. The most likely constraints and model parameters are estimated from the data *Y*. B. Mapping of genotypes to core pathways. The list of observed tumor genotypes is transformed to a list of altered core pathways by assuming that a pathway is altered if at least one member of that pathway is mutated. The order of core pathway alterations is then estimated using the H-CBN model. The influence of the gene-to-pathway mapping on the estimated constraints is analyzed by permuting genes among tumors (red arrows). To assess the stability of parameter estimates, bootstrap samples are drawn from the list of genotypes by sampling with replacement (green arrows) and the inference algorithm is run on each.

The H-CBN model has three layers (Figure 1A): (1) Mutations accumulate stochastically according to the partial order constraints. The rate at which each mutation arises and becomes detectable in the population is described by a parameter *λ_i_*. (2) The accumulation process is observed at the time of diagnosis, and the individual genotype *X* of a tumor contains all alterations that have occurred so far. (3) The observed mutation data *Y*, however, may differ slightly from the true genotypes, because of missing information or wrong interpretation. For example, intronic mutations, epigenetic silencing, or genomic deletions may not be detected, but have the same compromising effect on the gene. Conversely, recorded mutations may be passenger mutations with no functional consequences instead of drivers. These observation errors, which can "hide" the true genotypes, occur at rate ε. We estimate the accumulation rate for each mutation, the partial order constraints, and the error rate from the observed mutation data using maximum likelihood (ML).

The data for the H-CBN model is, for each tumor, a list of mutated genes or a list of altered pathways. Many mutations in different genes can have the same, or a similar, effect if they act in the same pathway [7], [32]. We model this phenomenon by analyzing twelve core pathways that were defined in ref. [8]. The core pathways are compiled from different annotation databases and describe the central signaling pathways altered in cancer. We assume that a core pathway is altered if any of its gene members has a non-synonymous mutation. We then use the H-CBN model to estimate in which order pathway alterations occur.

To assess the confidence of our estimates, subsets of the original data are repeatedly drawn with replacement (bootstrapped), the algorithm is applied to each, and the resulting output is analyzed (Figure 1B). A high similarity among bootstrap results indicates high confidence. A similar procedure is used to quantify the contribution of the gene-to-pathway mapping to the ordering. The mapping is expected to have an influence on the ordering, because some pathways are larger than others, and genes can be part of multiple pathways. To assess this effect, the genetic mutations are permuted between tumors, thereby breaking all correlations and leaving only those imposed by the mapping itself.

## Results

### Colorectal cancer

**Figure 2:**
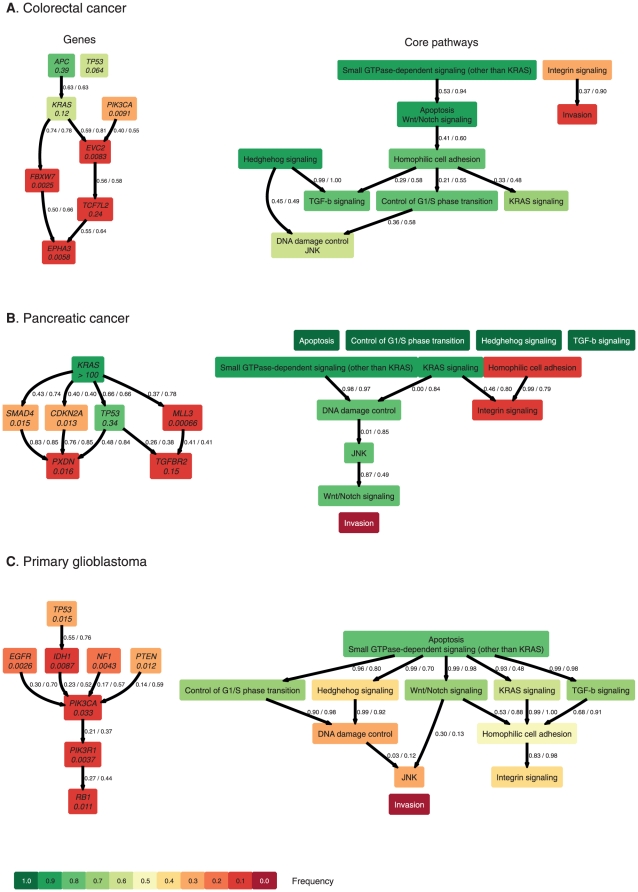
Most likely order constraints on the gene (left) and core pathway level (right) for colorectal cancer (**A**)**, pancreatic cancer** (**B**)**, and primary glioblastoma** (**C**)**.** Each edge in the graph denotes an order constraint on the accumulation of alterations. The two values labeling each edge and separated by a slash denote relative frequencies of occurrence of the order constraint in permutation and bootstrap samples, respectively. The estimated yearly accumulation rates are given below the gene name at each node of the graph. The color of a node reflects the frequency of the alteration (dark green 100% to dark red 0%). Nodes labeled with white font have frequencies of exactly 100% or 0% and were not considered for the statistical analysis.

The prevalence screen published by Wood et al. [10] contains data from 95 colorectal carcinoma samples in which the exons of 28 genes were sequenced. We considered non-synonymous changes as driver mutations. Eight genes that were found to have driver frequencies above 5% were chosen for estimating the gene-based order constraints, namely *APC* (82.1% frequency), *KRAS* (62.1%), *TP53* (56.8%), *PIK3CA* (24.2%), *FBXW7* (8.4%), *TCF7L2* (7.4%), *EPHA3* (5.3%), and *EVC2* (5.3%) (Table S1A). The estimated order constraints are displayed in Figure 2A. Mutations in the *APC* gene appear to be initiating, followed by mutations in *KRAS*, *PIK3CA*, and others. Interestingly, *TP53* is mutated independently of *APC* and *KRAS*, meaning that it could be mutated before or after these mutations. *APC* mutations have the highest accumulation rates (0.39 per year), in agreement with its early driving role in colorectal carcinogenesis. *KRAS* and *TP53* mutations accumulate at rates 0.12 and 0.06 per year, respectively. With the exception of *TCF7L2*, the remaining mutations have accumulation rates below 0.01 per year.

On average, 66% of the estimated order relations are also found in bootstrap samples. The average frequencies for each order constraint are displayed as edge labels in Figure 2A. The average false positive rate (FPR) of bootstrapped edges relative to the original estimate was 10.5% and the average false negative rate (FNR) was 33.6% (Table 1).

**Table 1:** Quality measures for the accuracy of estimated order constraints based on bootstrapping and permutations for genes and core pathways. FPR =  false positive rate, FNR =  false negative rate.

	Genes		Core Pathways				
	Bootstrap		Bootstrap		Permutations		
	FPR	FNR	FPR	FNR	FPR	FNR	
**Colorectal cancer**	0.105 (0,0.286)	0.336 (0,0.662)	0.098 (0, 0.235)	0.327 (0.053,0.684)	0.219 (0.098,0.333)	0.551 (0.211,0.842)	
**Pancreatic cancer**	0.151 (0,0.333)	0.427 (0.1,0.8)	0.136 (0,0.385)	0.154 (0,0.455)	0.085 (0,0.286)	0.519 (0.455,0.636)	
**Primary glioblastoma**	0.152 (0,0.179)	0.427 (0.221,0.75)	0.052 (0,0.133)	0.109 (0,0.286)	0.114 (0.067,0.182)	0.095 (0,0.19)	
**All Cancer Types**	-	-	0.04 (0,0.091)	0.061 (0,0.211)	0.164 (0.076,0.258)	0.289 (0.074,0.526)	

Values in parentheses denote the confidence intervals defined by the 5% and 95% quantiles.

After mapping colorectal cancer genes to core pathways, we found that Apoptosis and Wnt/Notch signaling pathways always occurred together. The same holds true for DNA damage control and JNK signaling. This situation arises from the specific set of genes predominantly altered in colorectal cancer, where multiple core pathways are hit by a single mutation, viz. *APC* for the Apoptosis/Wnt-Notch pathway and *TP53* for the DNA damage/JNK pathway (Table S1A). Because no further statistical inference on the order of identically hit pathways is possible, they were grouped together into two compound pathways (Figure 2A, right panel). Colorectal cancer progression begins with mutations in the Small GTPase pathway, followed by alterations of the Apoptosis/Wnt-Notch and Homophilic cell adhesion pathways, often caused by a single *APC* mutation. These alterations occur before perturbations of genes in the KRAS and TGF-b signaling pathways, as well as Control of G1/S phase. At later stages DNA damage control and JNK are altered (through *TP53*). Integrin signaling and subsequently Invasion are hit with the lowest frequencies, indicating a role at later stages of progression, but are modeled independently.

Under bootstrap re-sampling, the pathway FPR per relation is 9.8%, and the FNR is 32.7%, slightly smaller than the corresponding gene-based values. Under permutations, where all correlations of the genes are broken up, the FPR and FNR are 18.6% and 43.0%, respectively. Without the genetic correlations, the FPR is almost twice as large as the bootstrap FPR, i.e., more relations that were not in the original estimate were found. Also the FNR is higher under permutations as compared to the bootstraps, indicating that more of the original relations were missed. Together, these results demonstrate that the structure of the estimated model is sensitive to the particular combination of the genetic mutations in each tumor, and not only a simple consequence of the mapping.

To assess the impact of individual genes on the pathway-level results, we computed the likelihood of the model if a gene is left out from the analysis. Genes with a strong influence on the model fit also have an effect on the likelihood (Figure S1A). Genes with a strong impact were *APC, KRAS, PIK3CA*, and *TP53*, but also *TCFL7* and *MMP2* showed a recognizable effect.

### Pancreatic cancer

In the prevalence screen by Jones et al. [8], 22 genes in 90 cases of pancreatic cancer were sequenced. The most frequently (>4%) mutated genes were *KRAS* (98.9%), *TP53* (84.4%), *SMAD4* (25.6%), *CDKN2A* (24.4%), *TGFBR2* (6.7%), *MLL3* (5.6%), and *PXDN* (4.4%).

The genetic order constraints are displayed in Figure 2B. Mutations in *KRAS* initialize progression, followed by *TP53*, *CDKN2A*, and *MLL3*. *SMAD4* mutations occur independently of those in *KRAS*. The average FPR and FNR were 15.1% and 42.7%, respectively (Table 1). The estimated accumulation rate of *KRAS* mutations is very high, because the prevalence reaches almost 100%. *TP53* has the second highest rate of 0.34 per year, underpinning its central role in cancer progression. The other genes have lower accumulation rates between 0.15 (*TGFBR2*) and 6×10^−4^ (*MLL3*) per year.

On the core pathway level, the Apoptosis, G1/S transition, Hedgehog, and TGF-beta signaling pathways were altered in all 90 cases, and can thus be considered to occur at the earliest stages (Table S1B). Conversely, the Invasion pathway was never affected and may be assigned to the latest stage, if relevant at all.

The second stages of pancreatic cancer progression were found to be Small GTPase-dependent signaling and KRAS signaling, which arise independently. Both occur before a series of events, consisting of DNA damage control, JNK, and Wnt/Notch signaling. Integrin signaling is altered at late stages, after Homophilic cell adhesion mutations.

The stability of the pathway constraints was again higher as compared to the gene level. The FPR and FNR were 13.6% and 15.4%, respectively, under bootstrap sampling (Table 1). As in the case of colorectal cancers, the pathway FPR is slightly smaller than the genetic FPR (*P* = 3×10^−1^), and FNR is significantly smaller for pathways than for genes (*P* = 1.6×10^−15^). The FPR and FNR under permutations were 8.5% and 51.4%. Thus, on average half of the original relations is lost if the correlations of the genes are erased, indicating that they cannot be explained by the gene-to-pathway mapping alone. When testing for the influence of individual genes, *KRAS* and *TP53*, but also *PXDN* and *MYH2* had an appreciable effect on the likelihood (Figure S1B).

### Primary glioblastoma

Parsons et al. [9] sequenced 16 different genes in 83 glioblastoma cases. The most frequently (>5%) mutated genes were *TP53* (28.9%), *PTEN* (26.5%), *EGFR* (15.7%), *NF1* (15.7%), *PIK3CA* (9.6%), *IDH1* (8.4%), *PIK3R1* (7.2%), and *RB1* (7.2%). For this cancer type, the distribution of frequencies was more uniform, with less pronounced ‘mountains’ (genes mutated at high frequency), as compared to colorectal and pancreatic cancer. Of the 83 glioblastoma cases, five were of the secondary type, which is characterized by distinct genetics [9], [33]. Consequently, the secondary glioblastomas were found to have a significantly lower likelihood in a model fitted only on the remaining cases (*P*<0.01, cross-validation), indicating a worse fit for secondary glioblastomas and diverging mutational pathways. We therefore restricted the analysis to the remaining 78 primary glioblastomas. Of note, 16 out of these 78 cases contained mutations in none of the 16 genes sequenced.

The first mutation occurs in *TP53,* parallel to which *EGFR*, *NF1*, and *PTEN* mutate (Figure 2C). These alterations are followed sequentially by mutations in *PIK3CA*, *PIK3R1*, and *RB1*. The bootstrap stability of the relations was 11.6% (FPR) and 40.6% (FNR), respectively (Table 1). In line with the low frequencies of individual mutations, the estimated accumulation rates are all of order 0.01 per year or lower, showing that the probability for a specific gene alteration in this cancer type is low.

Despite the low frequencies of single gene mutations, the Apoptosis and Small GTPase core pathways were altered in 79.2% of the samples and are hit first (Figure 1C). As in the case of pancreatic cancers, no mutations were found in the Invasion pathway consistent with a late role (Table S1C). Among other early-mutated pathways are G1/S phase transition, Wnt/Notch signaling, and KRAS signaling. These pathways occur before mutations in DNA damage control and JNK signaling, as well as in Homophilic cell adhesion and Integrin signaling which form an independent branch.

The bootstrap stability of the relations was very high with a FPR of 5.2% and a FNR of 10.9% and therefore more stable than that determined on the gene level (*P* = 3×10^−20^ and *P* = 3×10^−30^, respectively). This compares to a permutation stability of 11.4% and 9.5%, respectively. Genes with a visible influence on the model fit were *TP53*, *RB1*, *PIK3CA*, *EGFR*, and *NF1* (Figure S1C).

### All Cancer Types

**Figure 3:**
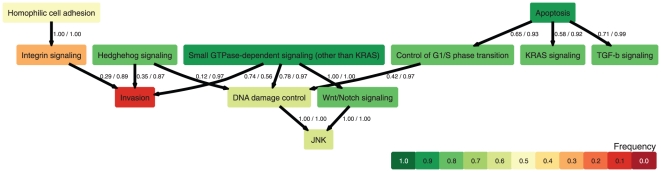
Global pathway progression model for all three cancer types. Each edge denotes an order constraint. The two numbers at each edge are the frequencies at which the given relation is observed under permutations of the genes and bootstrapping of the data, respectively. Colors denote the relative frequencies at which each pathway is hit by at least one mutation.

We integrated all 268 cases to estimate a "global" model of cancer progression on the pathway level. The resulting graphical representation is shown in Figure 3, the complete gene-to-pathway mapping can be found in Table S1D. The first event is Apoptosis which occurs before TGF-b and KRAS signaling, as well as Control of G1/S phase transition. Independently of this, Small GTPase-dependent signaling, Hedgehog signaling, and Homophilic cell adhesion are hit. Late events occurring after these changes are DNA damage control, Wnt/Notch signaling, and JNK, but also Integrin signaling and Invasion at late stages.

The model fit is highly stable with average FPR = 0.04 and FNR = 0.061 under bootstrapping. These values are significantly lower under bootstrapping than under permutations (FPR = 0.164, *P*<6×10^−50^, and FNR = 0.289, *P*<3×10^−35^, *t*-tests). The likelihood of the model is largely influenced by mutations in *TP53, KRAS, NF1, PIK3R1, PIK3CA, PTEN, RB1,* and *APC* (Figure S1D).

When comparing the topology of this model with the specific cancer cases, one finds that for colorectal cancer, 14 of 31 relations present in the colorectal poset are present in the unified model. Moreover, the global model contains 5 of 11 relations of the pancreatic poset and 5 out of 12 relations from the primary glioblastoma model. The constraints found in the global model may therefore be seen as the maximal subset of order constraints that hold true for all cancer types. The unified model, however, also contains constraints that could not be resolved in individual cancer types, where some pathways were grouped together, or have been either completely absent or universally present.

## Discussion

In this work we have analyzed the order constraints under which genetic alterations accumulate during cancer progression. We have studied the progression of colorectal and pancreatic cancer as well as glioblastomas on the level of individual genes, and also on the phenotype level of functional pathways. The analysis was based on a probabilistic graphical model, termed H-CBN, which was used to estimate the most likely set of order constraints, the rate at which each individual alteration occurs, and the observation error rate. We found differences in the ordering across cancer types and generally stronger evidence for order constraints on the pathway level than on the gene level.

In the gene-level model of colorectal cancer progression, *APC* has an initializing role, followed by mutations in *KRAS*. This resembles the progression proposed in an early study of this cancer type [19]. It appears, however, that the role of *TP53* mutations might be more flexible. In the original model, *TP53* mutations occurred after *KRAS* mutations. The late occurrence of p53 mutations has been experimentally observed since that time [34] and the most likely mutational pathway in our model also begins *APC* → *KRAS* → *TP53* → *PIK3CA*, followed by other mutations. However, the model we obtained allows for several other sequences of mutations and, on a population level, we estimate *TP53* mutations to occur independently of all other mutations (*P* = 0.01 in bootstrap samples), including *APC* and *KRAS*. Whether this difference reflects a limitation of the model, lack of statistical power, a defect in the experimental data, or our understanding of the pathways through which p53 acts, remains to be determined.

For the majority of genes the estimated accumulation rates of individual mutations *λ_i_* were of order 0.01 to 0.001 per year, but some mutations in colorectal carcinoma and pancreatic cancer have higher values of order one per year. Among those were *TP53*, *KRAS*, and for colorectal cancer, also *APC*. The inferred accumulation rates can be related to the fitness surplus a mutation contributes in a clonal expansion model. Under the assumption of an identical mutation rate per gene, an accumulation rate of 1 per year corresponds approximately to a fitness surplus of 2.6% (Methods Section). Therefore the high accumulation rates of *TP53*, *KRAS*, and *APC* can be explained by a fitness effect on the order of a few percent, compared to other mutations with lower fitness gains of order 10^−3^ or 10^−4^. Thus, these critical genes, which also form the mountains in the mutation landscape [8], [10], [20], may act as ‘superdrivers’ that provide a higher fitness gain than other genes.

Interestingly, this effect was not as pronounced in glioblastomas, where the mutation spectrum was also found to be flatter and fewer mutations were found in each tumor. One possible explanation for the absence of mountains in glioblastoma could be that the cellular context limits the possible fitness gain by single mutations, leading to a flatter mutation landscape. It may also be that other lesions that were not assessed in our data sets drive disease progression. It is striking, however, that despite the low levels of individual gene mutations and the absence of a clear genetic pattern there exist a robust signature of progression on the pathway level. Possibly, for glioblastoma larger parts of the pathways are active and vulnerable to mutations than in other cancer types that only permit mutations in a subset of genes with high mutation prevalence.

In pancreatic cancer, *KRAS* mutations are present in virtually all tumors. In this case, the estimated accumulation rate represents an almost instantaneous appearance of *KRAS* mutations at the onset of disease because only one case without *KRAS* is observed. It appears that the progression is initiated by *KRAS* mutations, followed by additional mutations in *CDKN2A*, *SMAD4*, *TP53*, and *MLL3* in a second stage and *PXDN* and *TGFBR2* in a third stage. Notably, the three stages are consistent with a three rate-limiting steps model fitted to age-incidence data [35].

The average frequency at which any pathway was altered in all samples was 70.0% in colorectal carcinoma, 72.0% in pancreatic cancer, and 67.7% in glioblastoma. This relatively high frequency probably reflects that the analyzed samples were all late-stage carcinomas. This finding is in contrast to the fact that, on the gene level, typically only a few genes have prevalence higher than 50%. Due to the joint action of multiple genes in different pathways, however, the frequency of pathway alterations can be high. A striking example is glioblastoma, were no gene has a mutation frequency higher than 35.8% (*TP53*), but the Apoptosis and Small GTPase pathways contain mutations in 79.2% of the samples analyzed.

The global model contains in total 19 order constraints among the 12 core pathways, which restrict the number of possible sequences in which the pathway alterations arise in a particular tumor from 12! = 479,001,600 to 356,640. Of these, the most likely sequence given the order constraints and the individual accumulation rates is: Apoptosis → TGF-b signaling → Small GTPase-dependent signaling (other than KRAS) → Wnt/Notch signaling → Control of G1/S phase transition → KRAS signaling → Hedghehog signaling → DNA damage control → JNK → Homophilic cell adhesion → Integrin signaling → Invasion. Mutations in the Apoptosis core pathway are early events in all cancer types evaluated here. For colorectal carcinogenesis, it is thought that *APC* mutations initiate carcinogenesis by compromising apoptosis in colonic endothelial cells resulting in the creation of adenomatous polypes [36]. In these cell pools, additional mutations accumulate and drive malignancy [37]. Our analysis shows that similar mechanisms could also act in pancreatic cancer and primary glioblastoma. The reason why the loss of apoptotic control is a critical step for initiating cancer can be understood from an evolutionary argument: The larger the population, the higher the number of cells at risk of acquiring additional mutations, and the higher the probability that cells with increased proliferation reach fixation [14]. In healthy organs, tissue organization reduces the number of cells at risk, for example, by organizing the tissue into stem cell niches such that only mutations in the stem cells can reach fixation [2]. This organization, however, breaks down if cells fail to undergo apoptosis and accumulate in an uncontrolled manner. In contrast to the findings for the Apoptosis pathway, mutations in the Invasion pathway are rare and occur late. This finding also agrees with our current understanding of carcinogenesis, wherein the capacity to invade other tissues and to metastasize is the last and often lethal step.

Despite the success of this model to recapitulate some critical aspects of neoplasia, there are several limitations. One is that our current annotation of signaling pathways is far from complete and many concurring definitions exist. We have therefore used a curated compilation of ‘core pathways’ from different sources to avoid a bias to a particular database. Yet the underlying data is often obtained from other species and cell types, whereas it is becoming increasingly clear that gene networks operate in a tissue- and species-specific fashion. It is striking, however, that despite these limitations in pathway annotations, our model is capable of inferring an ordering of alterations that is consistent with our current understanding of disease progression. As our knowledge of pathways improves through advanced systems biology approaches, we can expect our model to be more accurate and informative.

The robustness of the model fits was determined by bootstrapping, with estimated per-relation error rates of order 10%. In the future, the stability can be expected to improve further using larger cohorts from current cancer genome projects that aim to identify alterations in all genes in thousands of cancers. We observed that the stability of the genetic model was generally lower than that of the pathways. This may be attributed to the fact that, with a few exceptions such as *TP53* and *KRAS*, the mutation frequencies are very low. There is thus only weak evidence for specific order constraints on the accumulation of genetic mutations. This limitation should be at least partially resolved through the analysis of additional tumors, which will engender more confidence in the causality of mutations that occur at relatively low frequencies.

In our analysis, we considered a gene to be compromised in its function if there was a non-synonymous exonic mutation. While this may be true for most genes, there exist situations where this simple interpretation fails, or where additional lesions, such as genomic losses or epigenetic silencing, are required to inactivate a gene. These effects were subsumed in the error rate ε; larger data sets and integration of different data types can be expected to yield lower error rates and also better estimates of the order constraints.

In summary, the H-CBN model presents a framework for estimating the order constraints under which alterations in tumors accumulate over time. While there exist subtle differences in the order in which core pathways are altered in different cancer types, a common theme is that apoptosis is affected first, while invasion is affected at a late stage. The pathway H-CBN model may also be used to help define precise genetics-based progression measures with prognostic impact [25]. Moreover, the explicit nature of the error process allows for imputing the true genotypes from the data. This principle was shown to yield more accurate survival predictors for cytogenetic data for renal cell carcinomas [30]. The exomes of thousands of tumors will be sequenced in the next several years. We anticipate that the approach described here will be very useful for the analysis of these data and the statistical power gained from these large cohorts will further increase the accuracy of the model's predictions.

## Materials and Methods

### Hidden conjunctive Bayesian network model

We use the Hidden Conjunctive Bayesian Network (H-CBN) model defined in ref. [30]. In this model, alterations accumulate with respect to partial order constraints and the observed data may also contain observation errors. For example, errors can occur if a detected mutation is not functional, or if pathway membership is not correctly assigned. Genetic alterations occur according to exponential waiting time processes that are subject to partial order constraints [29]. The waiting time for mutation *i* is defined as
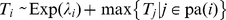
(1)


where 1/λ*_i_* is the average waiting time of the exponential distribution Exp, and the maximum is over all mutations *j* that are immediate predecessors of mutation *i* according to a fixed partial order *P*. The definition implies that all predecessor (or parent) mutations 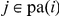 must be present before mutation *i* can occur.

The result of the waiting time processes is observed at the time of diagnosis, denoted *T_s_*, and the mutations that have occurred prior to diagnosis constitute the genotype of the tumor, *X* = (*X*_1_, …, *X*_n_), where *X_i_* = 1 if *T_i_*<*T_s_* indicates the occurrence of mutation *i*, and *X_i_* = 0 otherwise. Because the exact time of diagnosis with respect to the onset of tumorigenesis is unknown and likely to vary across patients, *T_s_* is modeled as an independent, exponentially distributed waiting time with parameter λ*_s_* = 1/(20 years). This choice was made to reflect the approximate time from the unknown onset of disease to diagnosis, which was estimated to be 10–20 years [35], [37]. The probability of a genotype *X* is thus given by: 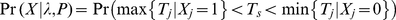
(2)


This probability can be computed efficiently by summing over all possible paths starting with zero mutations and leading to the genotype *X* under the constraints of the poset [29].

With probability ε the observation *Y_i_* of mutation *X_i_* is incorrect. Hence the probability of the observed genotype *Y* = (*Y*_1_, …, *Y_n_*), is given by



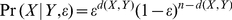
(3)


where *d*(*X*, *Y*) is the Hamming distance counting the number of differences between *X* and *Y*. The unobserved true genotypes *X* and the waiting times *T* = (*T*_1_, …, *T_n_*) are hidden variables in the Bayesian network defined by *T*, *X*, and *Y*. The resulting marginal probability of the data *Y* is given by



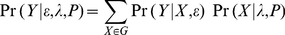
(4)


The symbol *G* denotes the lattice of genotypes compatible with the partial order *P*. The network parameters ε, λ = (λ_1_, …, λ*_n_*), and *P* are estimated using maximum likelihood (ML). For ε and λ, this involves an expectation-maximization algorithm. The most likely poset, denoted by 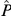, is found by simulated annealing [30]. A schematic overview of the H-CBN is given in Figure 1A.

### Genetic data and core pathways

Genetic data was obtained from the publicly available prevalence screens of refs. [8], [9], [10] in which the exons of 20 genes mutated at high frequency in each cancer case were screened for 100 patients. We considered a gene to be mutated if it contained at least one non-synonymous base substitution or indel. For estimating the genetic H-CBN model, only genes with mutation frequencies greater than 5% were selected for each cancer type. This pre-selection step was done for the genetic analysis because simulations show that the statistical power to learn relations for very rare mutations is low [30].

To assess progression on the pathway level, the complete genotypes with all recorded mutations were mapped to the set of twelve core pathways defined in ref. [8]. A given core pathway is assumed to be altered if at least one of its members is mutated. Some genes are members of multiple core pathways. If such a gene is mutated, all pathways in which the gene is active are considered to be affected. The process of mapping is illustrated in Figure 1B. The H-CBN software, and additional code and data for analyzing the gene-to-pathway mapping can be downloaded from our website http://www.cbg.ethz.ch/software/ct-cbn.

### Bootstrap and permutation analysis

To assess the robustness of the estimated order constraints, we performed a bootstrap analysis. For each cancer type, _100_ bootstrap samples were generated and the ML posets were estimated as described above. For each possible relation, the average occurrence in the ML poset was computed. Values close to one or zero indicate a high level of confidence for the presence or absence, respectively, of this relation. As overall measures of the stability of posets we consider the estimated false positive rate (FPR) and false negative rate (FNR) over all poset relations in the bootstrap posets 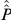 relative to the ML poset 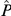 obtained from the original dataset:



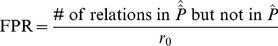
(5)




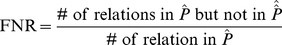
(6)


where *r*_0_ = *n*(*n*−1)/2 is the maximum number of relations (constraints) that can be found, which is the case for the linear poset. Here, the number of relations includes all direct constraints, termed "cover relations", and indirect constraints that can be derived from the cover relations. For example, the poset A → B → C, has two cover relations, which imply the indirect relation A → C, because C also comes after A, giving in total *r*_0_ = 3 relations.

The false positive rate is maximal, FPR = 1, if the bootstrap estimate 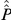 is linear, and all relations are different from those defined in 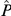 (e.g., by a reversal of the ordering, or if 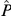 has no relations. If, on the contrary, all boostrapped relations are contained in the original poset, it follows that FPR = 0. The false negative rate FNR measures the relative number of relations in 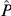 that are missed by 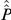. From the collection of bootstrap samples, the average FPR and FNR are computed.

To assess whether the estimated poset structures were merely a consequence of the pathway mappings and the marginal frequencies of the mutations, rather than an effect of their specific co-occurrence, we shuffled, for each gene, the occurrence of the mutations in order to break all correlations between mutations and again estimated the ML posets. The permutations hold the population frequencies of each mutation constant and generate the distribution of posets under the null hypothesis of independently occurring mutations. This procedure was repeated for 100 permutations and the frequencies of each relation in the ML poset were computed. We then computed FPR and FNR for the ML poset 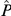 as in Eq. (6).

The differences between bootstrapping and gene-wise permuting are illustrated in Figure 1B. Bootstrapping is done on the set of tumors and the composition of each genotype remains intact. By contrast, gene-wise permutations break the correlation between genes, and therefore allow for assessing to which extent the observed dependencies on the pathway level stem from the definition of pathway membership alone.

### Relation to population genetics models

The average waiting time *τ_i_* for mutation *i,* given that all the necessary predecessors have occurred, is 1/λ*_i_*. This waiting time may be interpreted as the time until the mutation has occurred and reached fixation in the population. It has been shown recently that the expected waiting time between two successive clonal expansions in a Wright-Fisher model is approximately 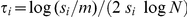 [14], [31]. Here, *s_i_* is the fitness of the clone, *m* the mutation rate, and *N* the population size. For 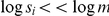, the approximate fitness surplus of mutation *i* from the rate λ*_i_* is



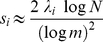
(7)


The average population size is assumed to be *N* = 10^6^ cells, and the mutation rate *m* = 10^−7^ per gene. The fitness depends only logarithmically on these two quantities, so even a change by an order of magnitude has only a moderate effect. Under these conditions, and for one cell generation per day, one has the relation 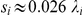, that is, fitness is approximately one fortieth of the yearly accumulation rate.

## Supporting Information

Figure S1 **Log-likelihood of the core pathway model after exclusion of individual genes for the three cancer types** (**A–C**) **and the global model including all cancer types** (**D**)**.** The solid line is the cumulative distribution function (CDF) of the log-likelihood under bootstrapping. Genes (black dots) with a strong influence on the likelihood are found in the tails of the CDF.
(EPS)

Table S1 **Gene-to-pathway mapping and alteration frequencies for all colorectal cancer (A), pancreatic cancer (B), primary glioblastoma (D), and the combined data for all three tumor types (D).**
(XLSX)
